# Calcium measurements in enzymatically dissociated or mechanically microdissected mouse primary skeletal muscle fibers

**DOI:** 10.1016/j.xpro.2023.102260

**Published:** 2023-04-30

**Authors:** Sonia Youhanna, Joseph Bruton, Kent Jardemark, Håkan Westerblad, Volker M. Lauschke

**Affiliations:** 1 Department of Physiology and Pharmacology, Karolinska Institutet, 171 77 Stockholm, Sweden; 2 Dr Margarete Fischer-Bosch Institute of Clinical Pharmacology, Stuttgart, Germany; 3 University of Tuebingen, Tuebingen, Germany

**Keywords:** Cell culture, Cell isolation, Microscopy, Biotechnology and bioengineering

## Abstract

Here, we provide a protocol for isolation of mouse primary skeletal muscle fibers using two alternative approaches—enzymatic dissociation or mechanical microdissection. We describe the procedures for surgical removal of muscle of interest and isolation of intact single-muscle fibers by either collagenase digestion or mechanical microdissection. We then detail intracellular calcium measurements by microinjecting or loading the isolated muscle fibers with membrane permeable calcium dyes. Finally, we outline steps for intracellular calcium quantification by fluorescent measurement.

For complete details on the use and execution of this protocol, please refer to Gineste et al.^1^

## Highlights

• Enzymatic dissociation or mechanical microdissection to isolate mouse muscle fibers
• Microinject or load isolated muscle fibers with membrane permeable calcium dyes
• Steps for intracellular calcium quantification by fluorescent measurement
• Microdissection preserves fiber microenvironment and maintains phenotypes and functions

## Abstract (editor highlights)

Publisher’s note: Undertaking any experimental protocol requires adherence to local institutional guidelines for laboratory safety and ethics.

## Graphical abstract

**Figure:**
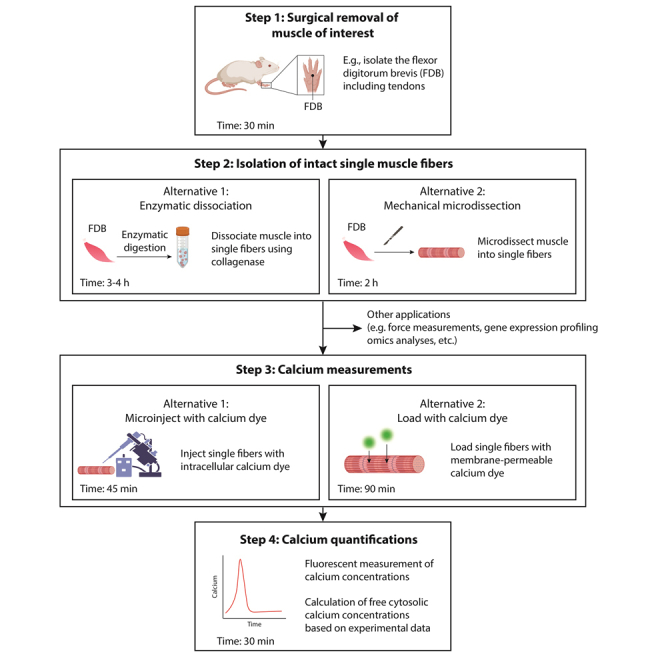

## Before you begin

Methods used for cell isolation may result in alterations of the extracellular matrix (ECM), which can impact cellular functionality and affect experimental outcomes in a wide variety of tissues, including liver,^2^ lung,^3^ bone^4^ and skeletal muscle.^5^^,^^6^ In this protocol, we describe two simplified methods for the isolation of single primary skeletal muscle fibers: 1) enzymatic isolation; and 2) surgical microdissection. We then detail how to use those isolated fibers for calcium measurements. The microdissection method results in improved maintenance of mitochondrial functionality, calcium handling and cellular transcriptomes. The protocol constitutes an adaptation and amalgamation of several enzymatic and microdissection techniques described earlier.^7^^,^^8^^,^^9^

### Institutional permissions

All animal experiments have to comply with local ethical guidelines and protocols. The protocols used here were approved by the Stockholm North Local Animal Ethics Committee and complied with the Swedish Welfare Ordinance, and applicable regulations and recommendations from Swedish authorities.

## Key resources table

**Table:** 

REAGENT or RESOURCE	SOURCE	IDENTIFIER
**Chemicals, peptides, and recombinant proteins**		

Collagenase type I	Sigma-Aldrich	C0130
Indo-1 pentapotassium, cell impermeant, 10 mM solution	Molecular Probes	I1202
Indo-1 acetoxymethyl (AM), membrane-permeable, salt	Invitrogen	I1223
Pluronic F-127	Sigma-Aldrich	P2443
DMSO	Sigma-Aldrich	472301
DMEM/F12	Thermo Fisher	21041025
Antibiotic-antimycotic solution (100×)	Thermo Fisher	15240096
Fetal bovine serum	Thermo Fisher	26140079
Tyrode solution	Thermo Fisher	J67607.K2

**Experimental models: Organisms/strains**		

Mouse (either sex, >5 weeks of age)	Janvier	C57BL/6JRj

**Other**		

Pen-type stimulation electrode	ADInstruments	MLA0320
Muscle fiber chambers	Aurora Scientific	1500A
Fluorescence system with two photomultiplier tubes	IonOptix	FSI-800
Surgical scissors (20 cm, straight)	Sigma-Aldrich	S3271
Micro-iris scissors	Albert Heiss	H-4260
Jeweller’s forceps	Sigma-Aldrich	F6521-1EA
SYLGARD™ 182 Silicone Elastomer Kit	Dow Corning	1673998
Dissection tray with silicone base	Home made	none
Drawing pins	Office Depot	12 mm
Aluminum	Sigma-Aldrich	GF08803616
Platinum	Sigma-Aldrich	267244-1.4G
Generic current pulse stimulator	World Precision Instruments	SYS-A300
Micropipette glass	Sutter Instrument Company, UK	Cat. no. BF100-58-10
Oil Hydraulic Micromanipulator	Narishige International Limited, UK	Cat. No. NMO-203

## Materials and equipment

• We herein describe the isolation of FDB muscles of 12 week old female C57BL/6JRj mice.

***Note:*** The protocol is also compatible with the isolation of FDB muscles and from other strains, ages or sexes. Furthermore, it can serve as the starting point for isolation of other murine skeletal muscles beyond the FDB. In such case, depending on the muscle of interest, slight protocol adaptations might be necessary.

• Complete Dulbecco’s modified Eagle medium (DMEM/F12): DMEM/F12 supplemented with 1% of the 100x antibiotic antimycotic solution containing penicillin (final concentration: 100 U/mL), streptomycin (final concentration: 100 μg/mL) and amphotericin B (final concentration: 0.25 μg/mL).
• Supplemented collagenase: Immediately before experiment, dissolve 0.3% (w/v) collagenase in complete DMEM/F12 at room temperature and supplement it with 20% fetal bovine serum. Use within 4 h of preparation.

***Note:*** Providers might not give an accurate measure of collagenase activity but activities around 280–345 U/mg should give optimal results.

• Prepare a 10x solution of Indo-1 AM and pluronic F-127 by dissolving them in DMSO as follows:

**Table:** 

Reagent	Final concentration	Amount
Indo-1 AM	0.5 mM	100 μg
Pluronic F-127	8 mM	20 mg
DMSO	–	200 μL
Total	–	200 μL

The solution is to be freshly prepared on the day of the experiment and kept in the dark at room temperature (20°C–25°C).

**Figure 1:**
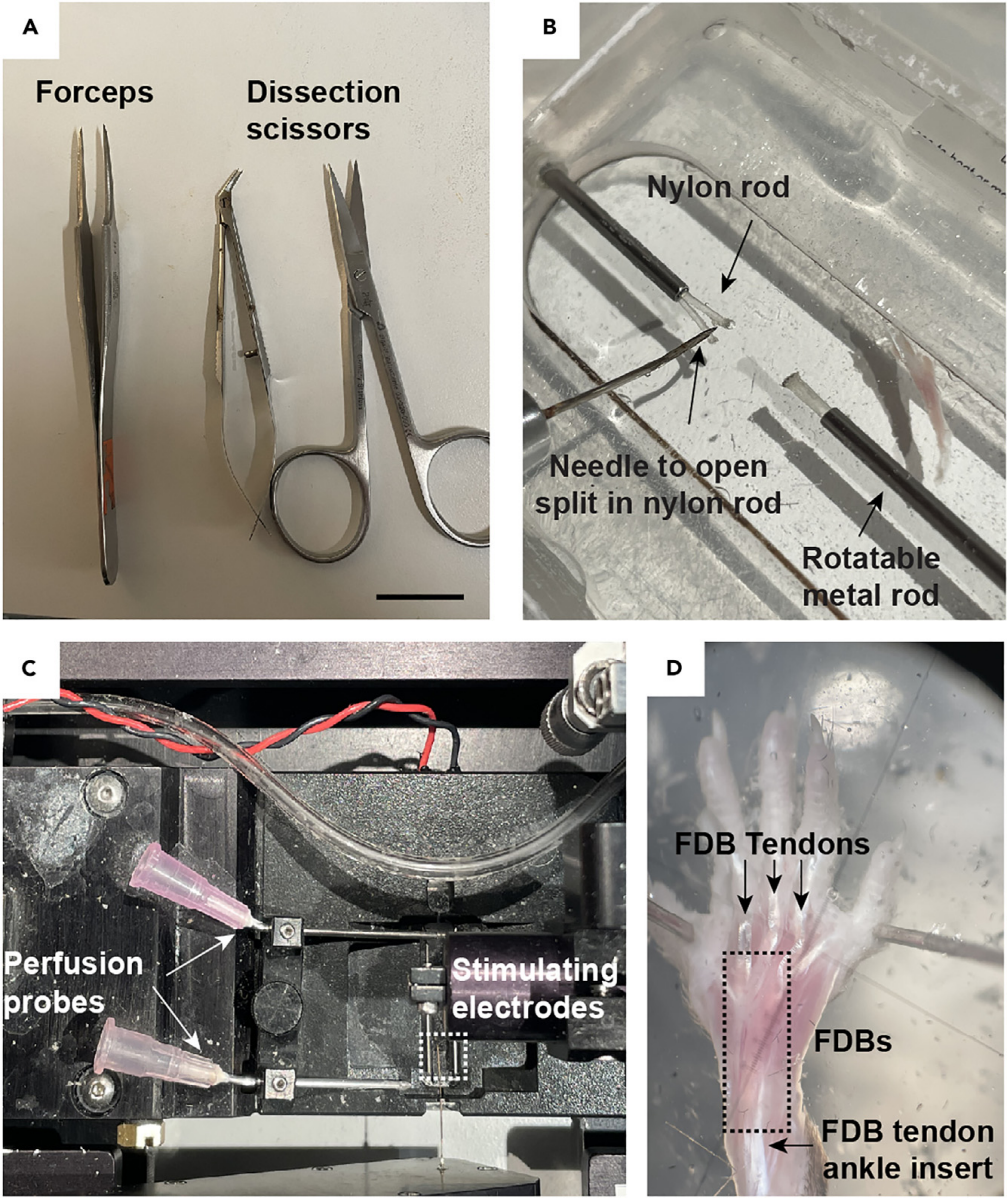
Equipment needed for isolation of FDB (A) Tools used during the dissection and the isolation process. Scale bar = 2 cm. (B) Image of the dissection tray with nylon rods. The procedure to open the split in the nylon rod where the proximal and distal tendons are inserted is shown. (C) Stimulation chamber with perfusion probes (arrows) and stimulation electrodes (dashed box). (D) Mouse paw with the FDB muscle exposed (dashed box). The FDB tendons and opposite ankle insert are shown.

***Note:*** Complete DMEM/F12 and Tyrode solution can be stored at 4°C for up to 1 week.

• Dilute the prepared 10x indo-1 AM/pluronic F-127 solution 1:10 in 1 mL DMEM/F12 or Tyrode solution in a closed Petri dish for incubation of the isolated flexor digitorum brevis (FDB) fiber. The solution is to be freshly prepared on the day of the experiment and should be kept in the dark.
• The experimental setup and required instruments for microdissection, including jeweller’s forceps, micro-iris scissors, dissection tray and stimulation chamber are shown in Figures 1A–1C.

## Step-by-step method details

### Part I. Surgical removal of FDB muscles

**Timing: 30 min**

This section describes the surgical removal of the FDB muscle, which serves as the substrate for the single fiber isolations described in the following part II.

1. Sacrifice the animal according to the locally approved procedures.
2. Remove the foot from the leg by amputating at the level of the ankle joint using sharp surgical scissors.
3. Place the foot in prewarmed Tyrode solution.
4. Pin the outermost toes of the isolated foot with dissection needles on the dissection pad with the plantar side of the foot facing upward (Figure 1D).

***Note:*** We use the lid of a pipette tip box coated with a 1 cm thick layer of Sylgard 182 elastomer as the dissection pad.

**Figure 2:**
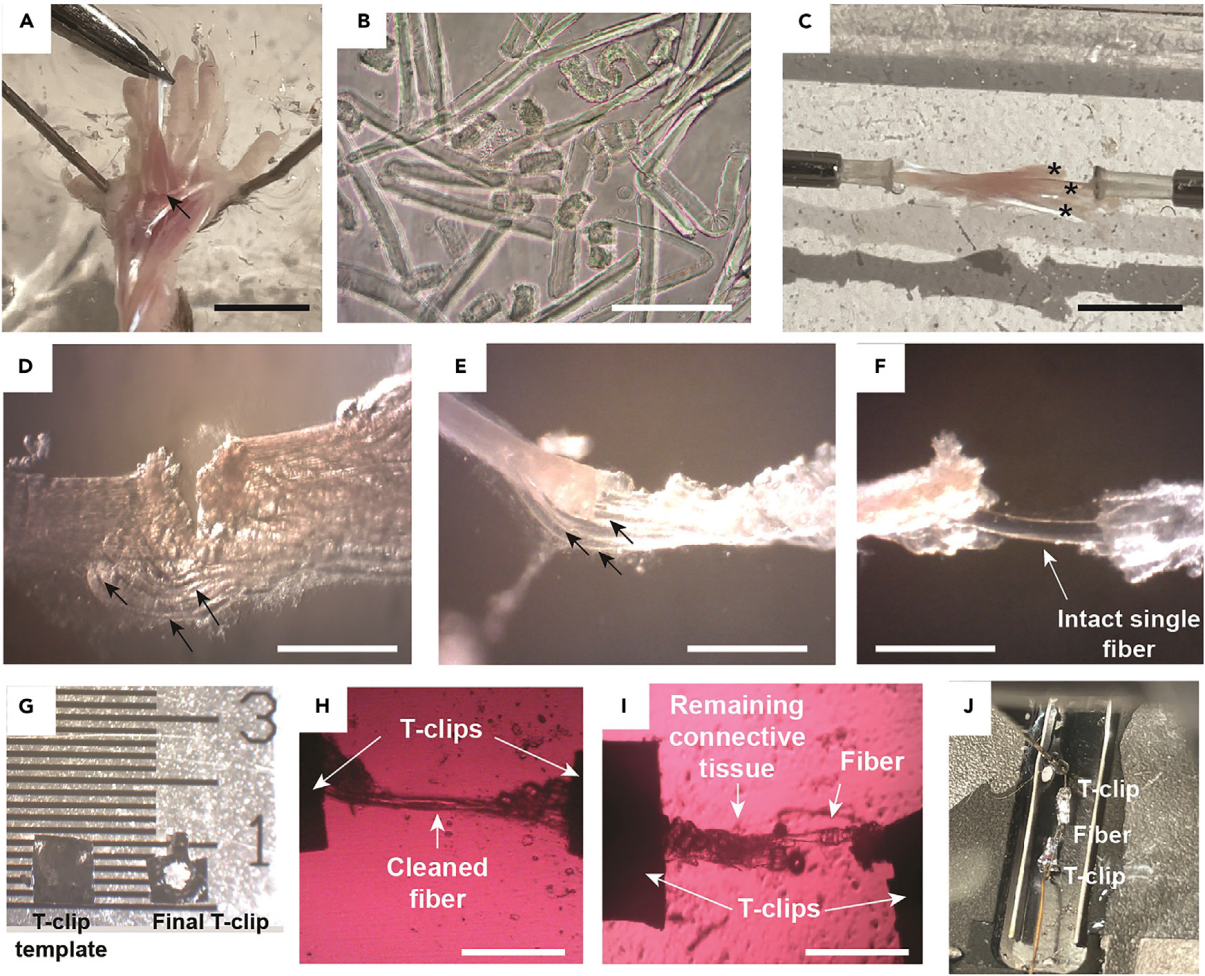
Illustration of critical steps during the mechanical single fiber dissection (A) Lifted FDB muscle (arrow). Scale bar = 1 cm. (B) Enzymatically dissociated fibers after 15 min attachment. Scale bar = 500 μm. (C) FDB muscle in the process of being separated into 3 digits (asterisks). The muscle is attached at both sides to the dissection tray via the nylon rods. Scale bar = 1 cm. (D) Several intact single fibers (arrows) attached to the tendons. Scale bar = 500 μm. (E) Single intact fibers after removal of damaged fibers and connective tissue. Scale bar = 500 μm. (F) Intact viable single fiber (highlighted by arrow). Scale bar = 500 μm. (G) T-clip template (left) and the final T-clip (right) are shown before attaching it to the single fiber. The labeled units on the ruler are in mm. (H and I) Clean single fiber (H) and, for comparison, a fiber with remaining connective tissue (I) are shown at 4x magnification. Scale bars = 500 μm. (J) Single fiber attached to T-clips and mounted in the stimulation chamber.

5. Remove the skin overlaying the FDB using dissection scissors.
6. Grip and lift the proximal tendon of the FDB using forceps.
7. Make a transverse incision to separate the FDB tendon from its origin at the back of the heel (calcaneus).
8. Carefully cut and remove all soft and connective tissue between the FDB muscle and the underlying flexor digitorum longus (FDL) using forceps and dissection scissors (Figure 2A).

**CRITICAL:** Always point the dissection scissors towards the FDL to minimize the risk of damaging fibers in the FDB.

9. Cut the FDB tendons as far out on the toes as possible to leave a long distal tendon. This helps a lot in the later stages of single fiber dissections.

**CRITICAL:** Make sure not to stretch the FDB muscle. This is best done by folding back the freed portion of the FDB onto the part of the FDB that remains joined by connective tissue to the underlying FDL muscle. Do not hold or pull the FDB upwards in the solution.

**CRITICAL:** During the whole procedure, ensure that the muscle does not dry out by covering the foot in warmed Tyrode solution.

### Part II. Isolation of single skeletal muscle fibers

Note that the following parts IIa and IIb are alternative methods for the isolation of single skeletal muscle fibers. For enzymatic dissociation, follow steps 10–18. For mechanical microdissection, follow steps 19–27.

#### Part IIa. Fiber isolation by enzymatic dissociation

**Timing: 3–4 h**

This part describes the isolation of primary single muscle fibers from FDB muscles by enzymatic dissociation.

10. Place 50 mL of complete DMEM/F12 in an incubator at 37°C.
11. Prepare 5 mL of supplemented collagenase type 1.
12. Clean FDB muscles of tendons, connective tissue, and blood vessels.
13. Incubate the cleaned muscle for 2–3 h at 37°C in the supplemented type 1 collagenase.
14. Transfer muscles to 3 mL fresh DMEM/F12 that was prewarmed in step 10.
15. Gently triturate the muscle using a regular blue plastic 1 mL pipette tip filled with 900 μL DMEM/F12.

***Note:*** Pipette slowly 10–20 times up and down to separate the individual muscle fibers.

***Note:*** One second up and one second down is the right timing.

16. Transfer a volume of 300 μL of the resultant muscle fiber suspension in laminin-coated 35 mm glass-bottom Petri dishes.
17. Allow fibers to attach for 15 min at 37°C (Figure 2B).
18. Add a further 2.7 mL of supplemented DMEM/F12.

***Note:*** Cells are ready for further experimentation up to 4 h after being attached in the Petri dish.

#### Part IIb. Fiber isolation by mechanical microdissection

**Timing: 2 h**

This part describes the isolation of primary single muscle fibers from FDB muscles by microdissection.

19. Place the FDB muscle in a custom-made dissection tray equipped with a pair of hollow movable metal 10G syringe needles mounted at opposite ends (see setup in Figure 1B).
20. Fix both tendons of the muscle by inserting the proximal and the distal tendons into the split ends of a nylon rod located inside the metal 10G syringe needles (Figure 2C).

***Note:*** The nylon rods are pulled into the needles to hold the tendons tightly and the needles can be rotated as needed when cutting away fibers and connective tissue during the dissection.

21. Make FDB fibers accessible by removing connective tissue, fat, and visible blood vessels using sharpened dissection forceps and scissors.
22. Separate the FDB muscle longitudinally into three FDB digits.

***Note:*** It is recommended to use a stereomicroscope with dark-field illumination with up to 40× magnification for this process.

**CRITICAL:** It is important to keep some muscle fibers of each FDB digit attached to the tendons at both sides (proximal and distal). These are the fibers that will be used for isolation.

23. Mount one digit in the dissection tray and fix into the split rods on both sides (Figure 2D).
24. Assess whether fibers are susceptible to electrical stimulation by applying single electrical stimuli using a pen stimulator at supramaximal voltage (≤10 V).

***Note:*** A brief twitch contraction of a few milliseconds followed by relaxation shows that the fibers are intact and functional (Methods video S1).

**CRITICAL:** We use a pen-type stimulation electrode connected to a generic current pulse stimulator with up to 100V stimulation voltage (World Precision Instruments; model no. SYS-A300 or Aurora Scientific; model no. 701C).

25. At 40× magnification, select a few twitching fibers that are positioned on the FDB surface and isolate those by cutting and removing all other fibers in small steps (Figure 2E).

**CRITICAL:** Check the remaining fibers repeatedly to identify fibers that give a robust contraction upon electrical stimulation.

**CRITICAL:** Make sure not to damage the fibers by overstretching the FDB digit.

26. Decide on one fiber and carefully remove the other fibers using the jeweller’s forceps and micro-iris scissors (Figure 2F).
27. Check that the isolated single fiber contracts upon electrical stimulation.

Method video S1. Electrical stimulation to confirm that fibers are intact and functional, related to step 24

### Part III. Calcium measurements in microdissected muscle fibers

For measurements of calcium in enzymatically dissociated fibers, we refer to the interested reader to the accurate methodological descriptions in ref.^10^^,^^11^ Note that the following parts IIIa and IIIb are alternatives for calcium measurements. In our experience, the best results are obtained with injection of the dye. For injection with intracellular calcium indicator, follow steps 28–34. For loading the fiber with a membrane permeable dye, follow steps 35–37.

#### Part IIIa. Injection with intracellular calcium indicator

**Timing: 45 min**

This section describes the measurement of calcium in isolated single fibers by microinjection of an intracellular calcium dye.

28. The proximal and distal tendons of the mechanically dissected single fibers are trimmed longitudinally and fitted into aluminum or platinum T-clips using two pairs of forceps to fold the T-clips.

**CRITICAL:** T-clips should be clamped to the tendon as close as possible to the muscle fiber as a long tendon can interfere with some downstream applications, such as force measurements (Figure 2G).

**CRITICAL:** It is essential to have an intact single fiber free of debris when using membrane permeable fluorescent calcium indicators, such as indo-1 AM or fura-2 dyes, because indicator trapped in remnant dead fibers can affect fluorescent measurements resulting in drastically increased experimental variability. For comparison, Figures 2H and 2I show a clean fiber and a fiber that was not well cleaned from connective tissue.

29. Install a micropipette in a micromanipulator and load it with ∼0.5 μL of 10 mM indo-1 salt solution.

***Note:*** If other calcium dyes are to be used, the concentration might need to be titrated for optimal results.

**CRITICAL:** Pressure during injection is driven by inert nitrogen gas.

**CRITICAL:** It is recommended to use Picospritzer gas pressure pulse settings of ∼100 psi with a duration of 1–5 ms.

30. Mount the fiber in the recording chamber between a force transducer and an adjustable holder (Figure 2J).

**CRITICAL:** During this procedure the stimulation chamber should be mounted onto an inverted microscope. Suitable stimulation chambers can be custom-made or are available commercially (e.g., Aurora Scientific; model no. 1500A). Suitable chambers have one mounting peg attached to a force transducer or fixed end and the other mounting peg attached to a movable screw. Superfuse the fiber with Tyrode solution at the desired temperature. For reference, the *in vivo* temperature of FDB muscles is 31°C (ref.^12^).

31. Adjust the stimulation current intensity and fiber length to obtain maximum tetanic force.
  a. To this end, determine the supramaximal stimulation intensity by stimulating with 70 Hz tetani at ∼1 min intervals and increase the voltage incrementally until the stimulation intensity is 10%–20% above that needed to achieve the maximum force.
  b. Thereafter, adjust the fiber length by using the movable screw to the length that gives maximum tetanic force.
32. Position the tip of the injection micropipette in close proximity to the fiber.

**CRITICAL:** The emitted fluorescence is measured with any suitable fluorescence system, which, for indo1 AM, has two photomultiplier tubes (e.g., HORIBA, Wedel, Germany or IonOptix, Amsterdam, The Netherlands).

**CRITICAL:** Measure and subtract background fluorescence of the fiber prior to injection by recording the emitted fluorescence signal values at 405 and 495 nm.

33. Inject the fiber with the dye.

**CRITICAL:** It is important to make sure that the fiber is in focus since imprecision can lead to an inaccurate estimate of the amount of injected dye.

**CRITICAL:** To control the amount of dye injected in the fiber, measure the increase in fluorescence signal at 405 nm–495 nm without stimulating the fiber. Withdraw the pipette when enough dye (∼two-times the background fluorescence) has been injected.

34. Allow an even distribution of the dye through the myoplasm of the fiber by waiting for at least 20 min before starting the experimental recordings.

#### Part IIIb. Loading the fiber with a membrane permeable dye

**Time: 1.5 h**

This section describes the measurement of calcium in isolated single fibers by loading the fiber with a membrane permeable indicator.

35. Incubate the fiber in the diluted indo-1 AM dye for at least 1 h at room temperature (20°C–25°C) to allow the dye to diffuse into the fiber.

**CRITICAL:** It is important to avoid that the fiber is drying out during loading in the stimulation chamber. This is done by placing a water filled cap from an Eppendorf tube in the incubation dish and ensuring that the lid is tightly sealed by wrapping it in Parafilm.

36. Mount the fiber as described above.
37. Superfuse with Tyrode solution for ∼30 min to remove any remaining dye outside of the fiber before starting the measurement.

#### Part IV. Quantification of calcium measurements

**Time: 30 min**

This part describes how intracellular calcium levels can be calculated based on fluorescent measurements of calcium indicators.

38. After the fiber is loaded with the dye using either the injection technique or the loading method, measure the fluorescence in the dark with excitation at 360 nm and dual emission at 405 and 495 nm.

**CRITICAL:** Always close the shutter for the excitation light when measurements are not performed to minimize light exposure of the dye-loaded fiber and prevent bleaching of the dye.

**CRITICAL:** Measure the emitted fluorescence of the dye with a suitable fluorescence system (e.g., HORIBA, Wedel, Germany or IonOptix, Amsterdam, The Netherlands).

39. Stimulate the fiber with supramaximal electrical pulses (0.5 ms in duration).
40. To obtain the steady-state [Ca^2+^]_cyt_ frequency, stimulate the fiber for 350 ms at 15–150 Hz every 1 min.
41. [Ca^2+^]_cyt_ is calculated using the ratio of light emitted at 405 nm to that at 495 nm (R) in the following equation^13^:

$${\left[{Ca}^{2+}\right]}_{cvt}=K_{d}*\beta *\left(R-R_{\min}\right)/\left(R_{\max}-R\right)\text{,}$$

with K_d_ as the apparent dissociation constant of the used dye, R_min_ as the ratio of 405 nm–495 nm emission at very low [Ca^2+^]_cyt_, R_max_ as the ratio of 405 nm–495 nm emission at saturating [Ca^2+^]_cyt_ and β as the ratio of the 495 nm signals at very low and saturating [Ca^2+^]_cyt_.

42. For an example of calculating [Ca^2+^]_cyt_ using the above equation in enzymatically dissected murine skeletal muscle we refer to the interested reader to ref.^14^

## Expected outcomes

The mechanical microdissection method allows isolation of 3 (or up to 6 if each toe is split longitudinally into two) viable single fibers from one FDB whereas as many as 50–100 viable fibers can be isolated when the enzymatic dissociation method is used. Isolated fibers deteriorate in functionality and phenotype, and we do not typically culture them for >24 h. The advantage of isolating intact skeletal muscle fibers by mechanical microdissection is that one can study both contractile function and calcium homeostasis of the muscle fiber within an intact microenvironment. Moreover, the use of single fibers permits metabolic profiling independently of hypoxia or nutritional diffusion limits as is the case when analyzing larger bundles or whole muscles.

## Limitations

The isolation of single skeletal muscle fibers by enzymatic dissociation does not preserve the native microenvironment, which impairs functional and metabolic outcomes.^1^ The limitations of microdissection are the technical sophistication and relatively low throughput.

## Troubleshooting

### Problem 1

Low viability of isolated single fibers (part I).

### Potential solution

Make sure to keep longer tendons. Always use the tips of the micro-iris scissors to cut away material. Use only forceps with undamaged tips that meet and close exactly.

### Problem 2

The fibers are not contracting (steps 24–27).

### Potential solution

Increase stimulating intensity. Alternatively, if fibers might be damaged during isolation or handling, dissect new muscle bundles.

### Problem 3

Difficulties in injecting indicator into the fiber (step 33).

### Potential solution

The micro-electrode might be clogged or blocked. Use a new micro-electrode. Increase the Picospritzer gas pressure and/or the duration of the injection pulses.

### Problem 4

Noisy fluorescence records (step 35).

### Potential solution

Increase the time for the indicator to load into the muscle fiber. Be careful to increase the loading time by no more than 15 min as you run the risk of overloading, which will give very smooth records but will incorrectly report time and amplitude of the calcium signals.

### Problem 5

Difficulty to visualize the calcium indicators after fiber injection (steps 38–40).

### Potential solution

Check that the fiber is illuminated with the excitation light. Replace the dye after >1 month of daily use and repeated freeze–thaw cycles.

## Resource availability

### Lead contact

Volker Lauschke (volker.lauschke@ki.se).

### Materials availability

All materials are commercially available as indicated.

## Data Availability

This study did not generate any unique datasets or codes.
